# Abnormal methylation mediated upregulation of LINC00857 boosts malignant progression of lung adenocarcinoma by modulating the miR‐486‐5p/NEK2 axis

**DOI:** 10.1111/crj.13765

**Published:** 2024-05-09

**Authors:** Haoyu Fu, Mingming Zhang, Xiaohui Liu, Yiming Yang, Ying Xing

**Affiliations:** ^1^ Department of Radiation Oncology Tangshan People's Hospital Tangshan China; ^2^ Department of Thoracic Surgery Tangshan People's Hospital Tangshan China; ^3^ Department of Breast Surgery Tangshan People's Hospital Tangshan China

**Keywords:** LINC00857, LUAD, metastasis, methylation, radiosensitivity

## Abstract

LINC00857 is frequently dysregulated in varying cancers, which in turn exerts carcinogenic effects; however, its DNA methylation status in promoter region and molecular mechanisms underlying the progression of lung adenocarcinoma (LUAD) remain rarely understood. Through bioinformatics analysis, we examined the expression state and methylation site of LINC00857 in LUAD and further investigated the properties of LINC00857 as a competitive endogenous RNA in the cancer progression. The current study revealed that the overexpression of LINC00857 in LUAD tissue and cells was mainly caused by the hypomethylation of the promoter region. LINC00857 knockdown prominently reduced cell proliferation, impeded cell migration and invasion, and restrained lymph node metastasis, with enhancing radiosensitivity. The effects of LINC00857 on tumor growth were also investigated in nude mice models. Subsequently, the downstream factors, miR‐486‐5p and NEK2, were screened, and the putative regulatory axis was examined. Overall, the regulatory effect of methylation‐mediated LINC00857 overexpression on miR‐486‐5p/NEK2 axis may be a new mechanism for LUAD progression.

## Graphical abstract

Low methylation of LINC00857 in the promoter region led to its high expression. Knockdown of LINC00857 restrained lymph node metastasis and stimulated radiosensitivity of LUAD cells. LINC00857 acted as a ceRNA, regulating NEK2 expression by binding to miR‐486‐5p.

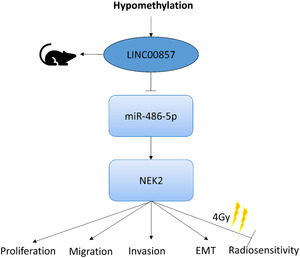

## 1 INTRODUCTION

Each year, at least one million deaths are caused by lung cancer, which is mostly responsible for deathrate associated with cancer globally. Lung adenocarcinoma (LUAD) is a prevalent histology.^1^ LUAD is found almost exclusively in peripheral lung tissue and arises from smaller airways.^2^ Surgery is suggested for patients with early LUAD, but most patients are suffering from an early relapse, in the first 2 years, after complete resection,^3^ which could be attributed to metastatic cascade in those patients.^4^ Hence, elucidating mechanism underlying the LUAD occurrence and metastasis is critical to provide better treatment strategies for LUAD.

Epigenetics is a major promising area within current biomedical fields.^5^ As a critical mechanism of epigenesis, DNA methylation is key in regulating gene expression, chromatin modification, genome imprinting, and stabilization.^6^ Abnormal DNA methylation is increasingly being associated with the pathogenesis of many human cancers.^6^ For example, in lung cancer, ADAMTS18 was hindered by promoter CpG methylation and enhanced cisplatin sensitivity through EGFR/AKT signaling.^7^ Hypomethylation‐induced upregulation of cancer/testis antigen KK‐LC‐1 promoted the LUAD development via activation of Notch1/Hes1 signaling.^8^ DNA methylation pattern of cancer cells impacts levels of protein‐coding genes and tumor‐related lncRNAs. Zhi et al. reported a computational strategy to re‐annotate Infinium 450 k array, with lncRNA promoter DNA methylation being closely related to lncRNA transcription.^9^ However, there are few studies on the DNA methylation status of lncRNA in LUAD.

LncRNAs are non‐coding RNAs without open reading frame (ORF) and are usually spliced and polyadenylated.^10^ Different subtypes of lncRNAs participate in modulating protein‐coding genes, but the specific molecular mechanisms are varied.^11^, ^12^, ^13^ Among the regulatory mechanisms, competitive endogenous RNA (ceRNA) mechanism has attracted widespread attention. lncRNA and miRNA might be a key in regulating of gene expression. LncRNA can harbor miRNA recognition elements by acting as a ceRNA.^14^ Numerous lncRNAs can modulate LUAD development through this mechanism. For example, lncRNA WDFY3‐AS2WDFY3‐AS2 facilitates development of LUAD via targeting miR‐491‐5p/ZNF703 axis.^15^ LncRNA FTX plays a carcinogenic effect in LUAD, regulating NUCB2 level via targeting miR‐335‐5p to stimulate malignant LUAD cell phenotype.^16^ Interaction of lncRNA‐SNHG7/miRNA‐181/cbx7 constrains LUAD progression.^17^ All above evidence provides an initial understanding that ceRNA regulatory network may exert a pivotal role in LUAD development.

LINC00857 is abnormally expressed in several cancers, which may impact on the tumor biology. For example, LINC00857 was upregulated in gastric cancer tissue and had positive association with low survival rate and tumor size. Knockdown of LINC00857 can induce dysregulation of cell cycle and thus repress the proliferation of gastric cancer cells.^18^ Similarly, LINC00857 was upregulated in bladder cancer tumor tissue, while its functional knockdown can remarkably reduce cell viability of bladder cancer by inducing apoptosis and make these cells sensitive to cisplatin.^19^ As uncovered by Liu et al.,^20^ in prostate cancer, the downregulation of LINC00857 was implicated in patients' prognoses. Expression and role of LINC00857 in LUAD have been extensively studied, but the specific mechanism underlying dysregulation of LINC00857 in LUAD is not completely understood, which awaits further research.

We sought to unveil function of epigenetic changes of LINC00857 in LUAD, its biological effects on LUAD tumor, and the possible molecular mechanism underlying the LUAD occurrence and metastasis. Our research may generate fresh insights into LUAD pathogenesis and offer a new perspective for treatment.

## 2 MATERIALS AND METHODS

### 2.1 Microarray analysis

The 450K methylation data (Table S1) (normal: 32, tumor: 475), miRNA expression data (Table S2) (normal: 46, tumor:521), and mRNA expression data (Table S3) (normal: 59, tumor: 535) of The Cancer Genome Atlas (TCGA)‐LUAD were obtained from TCGA (https://portal.gdc.cancer.gov/) database. The R package “MethylMix”^21^ was used to identify methylation driver genes (Table S4) (log |FC| > 0.5, FDR < 0.05, Cor < −0.3) while lncATLAS database (http://lncatlas.crg.eu/) was for analysis of subcellular location of LINC00857. lncBase (http://carolina.imis.athena-innovation.gr/diana_tools/web/index.php?r=lncbasev2%2Findex) database was applied to identify interaction between miRNA and LINC00857, and further starBase (http:/ /starbase.sysu.edu.cn/), miRDB (http://mirdb.org/), and mirDIP (http://ophid.utoronto.ca/mirDIP/index.jsp#r) databases were applied to screen regulatory targets downstream of miRNA. R package “edgeR” was introduced for differential analysis between normal and tumor groups for miRNA and mRNA expression data (log|FC| > 2.0, FDR < 0.05). Patients were assigned into high and low expression groups based on median value of target gene levels in tumor, and the impact of target gene expression levels on patient prognosis was studied through the R package “survival.” Pearson correlation analysis was completed among LINC00857 and mRNA and DNA methylation.

### 2.2 Collection of clinical tissues

Among the patients who underwent surgical resection of LUAD tumors at Tangshan People's Hospital, 50 pairs of matched LUAD and adjacent normal tissue were excised and frozen in liquid nitrogen immediately. Samples were included when met these criteria: (1) The excised samples were diagnosed as LUAD based on histopathological evaluation and examination; (2) the patients without any other treatment prior to surgery had provided an informed consent form; (3) definite pathological staging. The project was approved by the Research Ethics Committee of Tangshan People's Hospital.

### 2.3 Cell culture

Human normal lung epithelial cell line BEAS‐2B (BNCC359274) and LUAD cell lines A549 (BNCC337696), NCI‐H1299 (BNCC100268), PC‐9 (BNCC340767), H838 (BNCC100696), and NCI‐H23 (BNCC341422) were obtained from BeNa Culture Collection (China). They were kept in RPMI 1640 (Gibco, USA) containing 10% fetal bovine serum (FBS) and 100 U/mL penicillin–streptomycin (Invitrogen, USA) and kept at 37°C in an incubator with 5% CO_2_.

### 2.4 Plasmid construction and cell transfection

sh‐LINC00857 and its negative control (sh‐NC), oe‐LINC0087 and its negative control (oe‐NC), miR‐486‐5p mimic or inhibitor and their respective negative controls, NEK2 overexpression plasmids pcDNA3.1‐NEK2, and pcDNA3.1 (control) plasmids were all accessed from GeneChem (China). One day prior to transfection, LUAD cells were plated into six‐well plates. Transfection was done by Lipofectamine 2000 (Invitrogen, USA).

### 2.5 qRT‐PCR

GenElute™ Total RNA Purification Kit (Sigma‐Aldrich, USA) was employed to isolate total RNA from tissue or cells. In an effort to determine mRNA expression of LINC00857 and NEK2, PrimeScript™ RT kit (TaKaRa, Japan) was introduced for synthesis of cDNA. For miR‐486‐5p, reverse transcription was completed by miScript reverse transcription kit (Qiagen GmbH, Germany). Quantification was completed by qRT‐PCR analysis kit (Thermo Fisher Scientific, USA). GAPDH was applied as endogenous control of LINC00857 and NEK2, and U6 as endogenous control of miR‐486‐5p. The 2^−ΔΔCt^ method was utilized to compute relative gene levels normalized by GAPDH and U6. Primer sequences are listed in Table 1.

**TABLE 1:** The list of primers.

Gene	Forward (5′‐3′)	Reverse (5′‐3′)
qRT‐PCR		
LINC00857	CCCCTGCTTCATTGTTTCCC	AGCTTGTCCTTCTTGGGTACT
NEK2	TGCTTCGTGAACTGAAACATCC	CCAGAGTCAACTGAGTCATCACT
GAPDH	CCCATCACCATCTTCCAGGAG	CTTCTCCATGGTGGTGAAGACG
miR‐486‐5p	TGTACTGAGCTGCCCCGAG	CTCAACTGGTGTCGTGGAGTC
U6	CTCGCTTCGGCAGCACA	AACGCTTCACGAATTTGCGT
Sequences for MSP		
LINC00857 (methylated)	TTTAAGGTTAATGGTAGGGGTTATC	ATAAATAAAAAAACCGAAACTCGAA
LINC00857(unmethylated)	TAAGGTTAATGGTAGGGGTTATTGT	ATAAATAAAAAAACCAAAACTCAAA

### 2.6 Methylation‐specific PCR

We used MethPrimer 22 (http://www.urogene.org/methprimer/) to predict the CpG island in the LINC00857 promoter region and designed primers for quantitative methylation analysis. One microgram of genomic DNA was modified with bisulfite with Methylation‐Gold Kit (Zymo Research Corporation, USA). Unmethylated cytosine residues were converted to thymine, while methylated cytosine residues remained in the CpG site as cytosine following standard sodium bisulfite DNA modification. Results are presented in the form of electropherograms. Primer sequences are presented in Table 1.

### 2.7 Western blot

LUAD cells and tissue were lysed in a radioimmunoprecipitation assay lysis buffer supplemented with a protease inhibitor (Milipore, USA). Then, protein concentration was assessed with Dioctinic Acid Kit (Thermo Fisher Scientific, USA). The extracted proteins were separated in 12% SDS‐PAGE and then transferred to PVDF membrane (Millipore, USA). Membrane was sealed with 5% skimmed milk for 2 h. Then, membrane and the primary antibodies rabbit anti‐E‐cadherin (1:10,000, ab231303, Abcam, UK), N‐cadherin (1:5000, ab76011; Abcam, UK), and GAPDH (1:10,000, ab181602, Abcam, UK) were incubated together overnight at 4°C. Next, secondary antibody goat anti‐rabbit IgG H&L (1:2000, ab205718, Abcam, UK) was supplemented for 1‐h‐incubation at room temperature. Finally, the electrochemiluminescence kit (Pierce Biotechnology, USA) was implemented to visualize protein bands.

### 2.8 CCK‐8

Cell Counting Kit‐8 (Beyotime, China) was applied to test cell proliferation. Cells were plated into 96‐well plates (2 × 10^3^ cells/well). Then, 10 μL CCK8 reagent (Dojindo Molecular Technologies, Japan) was supplemented, and cells were maintained in under routine conditions for 2 h. Microplate Reader (Biorad, USA) was implemented to assess optical density of the cells at 450 nm after 0, 24, 48, 72, and 96 h of culture.

### 2.9 Cell migration and invasion assays

Migration was measured in Transwell inserts (8 μm pore size, CoStar, USA). Forty‐eight hours later, cells were resuspended in RPMI 1640 medium (200 μL) and then inoculated into serum‐free medium in the upper Transwell chamber, and DMEM + 10% FBS was placed in the lower chamber. For invasion assessment, Matrigel (Corning, USA) was diluted in serum‐free DMEM and inoculated into the upper chamber of an 8 μm pore size insert. Transfected cells were seeded into the upper chamber for invasion assay, and DMEM with 10% FBS was filled into the lower chamber. Following 24 h of culture at 37°C, cells remained in the upper chamber were discarded and cells in the lower chamber were fixed with 4% paraformaldehyde and stained with 0.1% crystal violet. Microscope (Olympus Corporation, Japan) was applied to count the number of migrated or invaded cells in five different fields of view.

### 2.10 X‐ray irradiation therapy

Transfected LUAD cells were plated into 24‐well plates overnight, follow in order treatment with X‐ray radiation (0, 2, 4, 6, and 8 Gy). Finally, cell survival was tested via CCK‐8 analysis.

### 2.11 Dual‐luciferase reporter gene assay

Fragments with LINC00857‐wild‐type (WT) or LINC00857‐mutant (MUT) were cloned into pmirGLO vectors (Promega, USA). The 3′‐untranslated region (UTR) of NEK2‐WT and corresponding NEK2‐MUT was cloned into the psi‐CHECK2 luciferase reporter vector (Promega, USA). Lipofectamine®2000 was applied to co‐transfect WT/MUT LINC00857 or NEK2 with miR‐486‐5p mimic or NC mimic into LUAD cells. At 48 h after transfection, luciferase activity was assayed by Dual‐Luciferase Reporter Assay (Promega, USA).

### 2.12 Nude mice assay

BALB/c nude mice (*n* = 30) aged 4 to 6 weeks were accessed from Shanghai SLAC Animal Center (China) for tumor proliferation assay and randomly assigned into two/three groups (6 mice/group). A549 cells were stably transfected with a lentiviral vector containing sh‐LINC00857 or sh‐NC (GeneChem, China), and transfected cells were subcutaneously xenotransplanted into back and limbs of BALB/c male nude mice. Tumor volumes were evaluated once a week and tumor growth curve was plotted. Calculation formula is: volume = length×width^2^ × 0.5. Five weeks after xenotransplantation, mice were sacrificed. Finally, tumors were isolated and the weights were measured.

In order to evaluate the resistance of LUAD in vivo, mice were divided into three groups (sh‐NC, sh‐NC + 4Gy, sh‐LINC00857 + 4Gy). Referring to previous studies,^22^ when the tumor grew to an average volume of about 100 mm^3^, the mice in the sh‐NC and sh‐LINC00857 groups were continuously exposed to radiation for 5 days. Tumor volume was measured every 5 days. The mice were sacrificed after 20 days. The tumor was separated and weighed.

### 2.13 Immunohistochemistry

The tumor tissue was prepared into paraffin sections. Different xylenes (xylene I, xylene II) and gradient ethanol were used for staining pretreatment. Then, EDTA antigen repair solution was used for microwave repair, and endogenous catalase blockers were dripped to block it. Next, it was incubated with Ki67 antibody (ab15580, Abcam, UK) at 4°C overnight, followed by incubation with secondary antibodies at room temperature for 1 h before being observed with a diaminobenzidine substrate (Sigma, USA). The immune tissues were observed and photographed using a fluorescence inverted microscope.

### 2.14 Statistical analysis

Statistical analysis was performed on SPSS 22.0 (IBM Corp. Armonk, USA) and GraphPad Prism 6.0 Software (GraphPad Inc., USA). All data from three repeats of each experiment were presented as mean ± SD. Comparison in two groups was tested by *t* test, and comparison of multiple groups was tested via one‐way ANOVA. Pearson correlation analysis was applied to assess correlation between various genes. *P* < 0.05 represented that difference was statistically significant.

## 3 RESULTS

### 3.1 LINC00857 is highly expressed in LUAD

**FIGURE 1:**
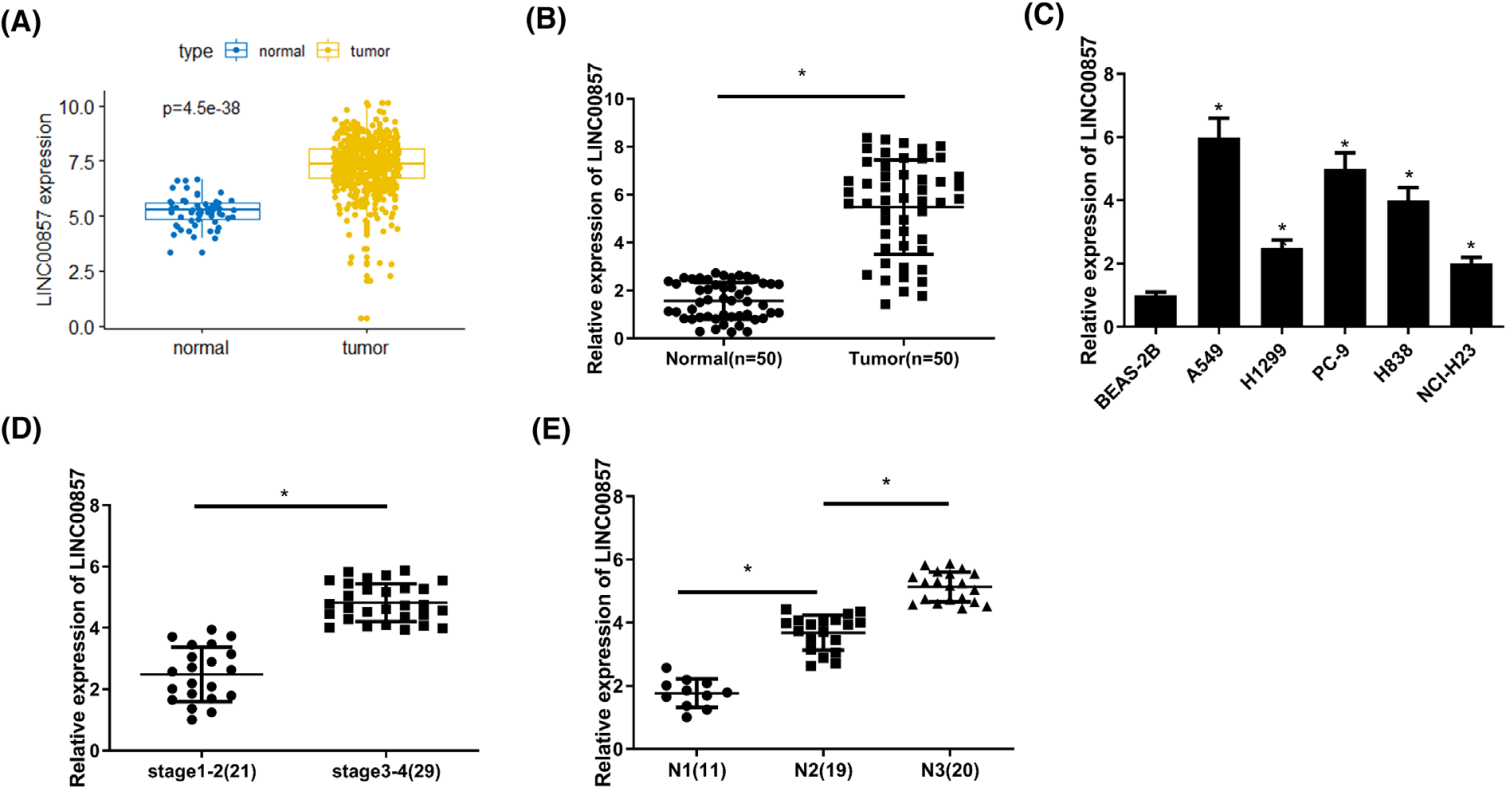
LINC00857 is highly expressed in LUAD. (A) Box plot of LINC00857 expression in the TCGA‐LUAD database, blue represents normal tissue while yellow represents tumor tissues; (B) LINC00857 expression in clinical LUAD tissue and adjacent normal tissue; (C) the expression of LINC00857 in human normal lung epithelial cells and human LUAD cells; (D) the relationship between LINC00857 gene expression and tumor clinical stage; (E) the relationship between LINC00857 gene expression and N stage; **P < 0.01, ***P < 0.001, ****P < 0.0001.

In the purpose of revealing LINC00857 expression in LUAD, as revealed by bioinformatics analysis, LINC00857 level in tumor tissue was prominently upregulated compared with the paired normal one in TCGA database (Figure 1A). LINC00857 level was detected in clinical LUAD tissue and matched para‐cancerous tissue, as well as cell lines (BEAS‐2B, A549, H1299, PC‐9, H838, and NCI‐H23), through qRT‐PCR, and we uncovered that LINC00857 level in LUAD tumor tissue was markedly higher than in corresponding para‐cancerous tissue (Figure 1B). In comparison to normal lung epithelial cells, LINC00857 level was substantially higher in LUAD cells (Figure 1C). Combined with the analysis of 50 clinical patients, LINC00857 level was elevated with increasing tumor stage and N stage (Figure 1D,E). Hence, LINC00857 was highly expressed in LUAD.

### 3.2 Knockdown of LINC00857 represses malignant progression of LUAD cells

**FIGURE 2:**
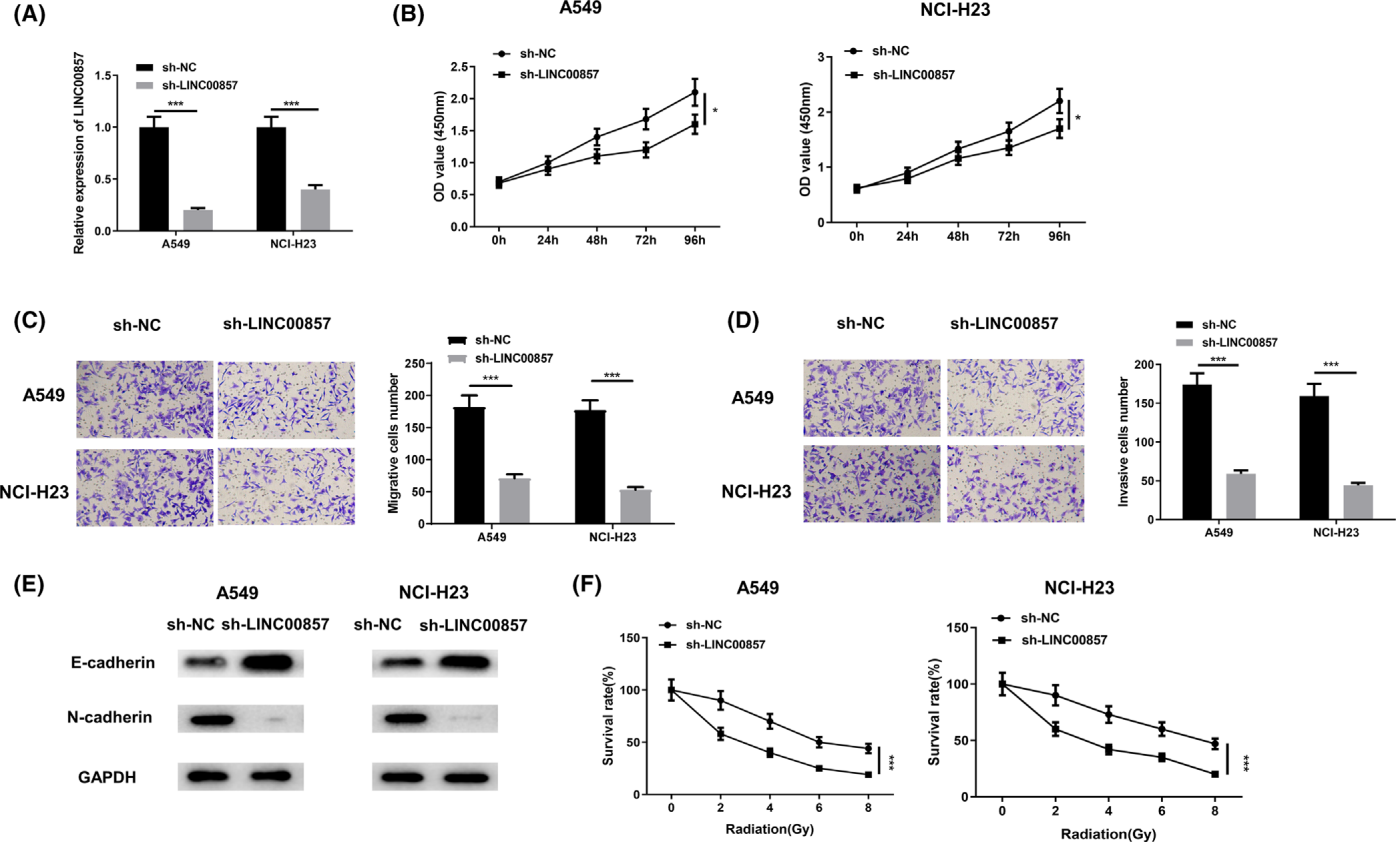
Knockdown of LINC00857 represses malignant progression of LUAD cells. (A) A549 and NCI‐H23 cells were transfected with sh‐NC or sh‐LINC00857, and the transfection efficiency was detected by qRT‐PCR; (B) CCK‐8 assay was used to detect the viability of A549 and NCI‐H23 cells transfected with sh‐NC or sh‐LINC00857; (C, D) Transwell assay was applied to detect the migration and invasion of A549 and NCI‐H23 cells transfected with sh‐NC or sh‐LINC00857 (100×); (E) Western blot was employed to detect the effect of downregulation of LINC00857 on the expressions of EMT markers E‐cadherin and N‐cadherin; (F) different intensities of X‐ray radiation treatment was used for proliferation determination of A549 and NCI‐H23 cells transfected with sh‐NC or sh‐LINC00857; *P < 0.05, ***P < 0.001.

In an effort to study biological role of LINC00857 in LUAD progression, cell transfection was done. As LINC00857 level being the highest in A549 cells but the lowest in NCI‐H23 cells, these two cell lines were applied for further assays after knockdown, so as to achieve the best transfection efficacy. Cells transfected with sh‐NC were applied as a control and sh‐LINC00857 was transfected into A549 and NCI‐H23 cells. Following transfection of sh‐LINC00857, LINC00857 level in both cell lines reduced noticeably as tested by qRT‐PCR, indicating well transfection efficacy in support of their usage for subsequent assays (Figures 2A and S1A). CCK‐8 analysis indicated that silencing of LINC00857 greatly restrained viability (Figure 2B). Transwell analysis uncovered that inhibition of the expression of LINC00857 remarkably reduced cell migratory and invasive capabilities (Figure 2C,D). The effect of downregulation of LINC00857 on EMT marker expression (E‐cadherin and N‐cadherin) was also tested through western blot. We uncovered that the downregulation of LINC00857 boosted E‐cadherin level and repressed N‐cadherin level, thus repressing EMT process of LUAD cells (Figure 2E). In addition, transfected cells were treated with infrared radiation (IR), and then evaluated cell viability. In comparison to LUAD cells with sh‐NC, survival rate of the cells with sh‐LINC00857 was remarkably reduced (Figure 2F), indicating an enhanced radiosensitivity of the cells. Hence, knockdown of LINC00857 restrained proliferation, migration and invasion of LUAD cells and facilitated radiosensitivity. In addition, we examined the effects of LINC00587 overexpression on LUAD cell function and radiosensitivity. The results showed that overexpression of LINC00587 further enhanced the proliferation, migration and invasion of LUAD cells, as well as the EMT process (Figure S2A–E), and reduced the radiosensitivity of LUAD cells (Figure S2A–F).

### 3.3 The upregulation of LINC00857 is caused by hypomethylation in the promoter region

**FIGURE 3:**
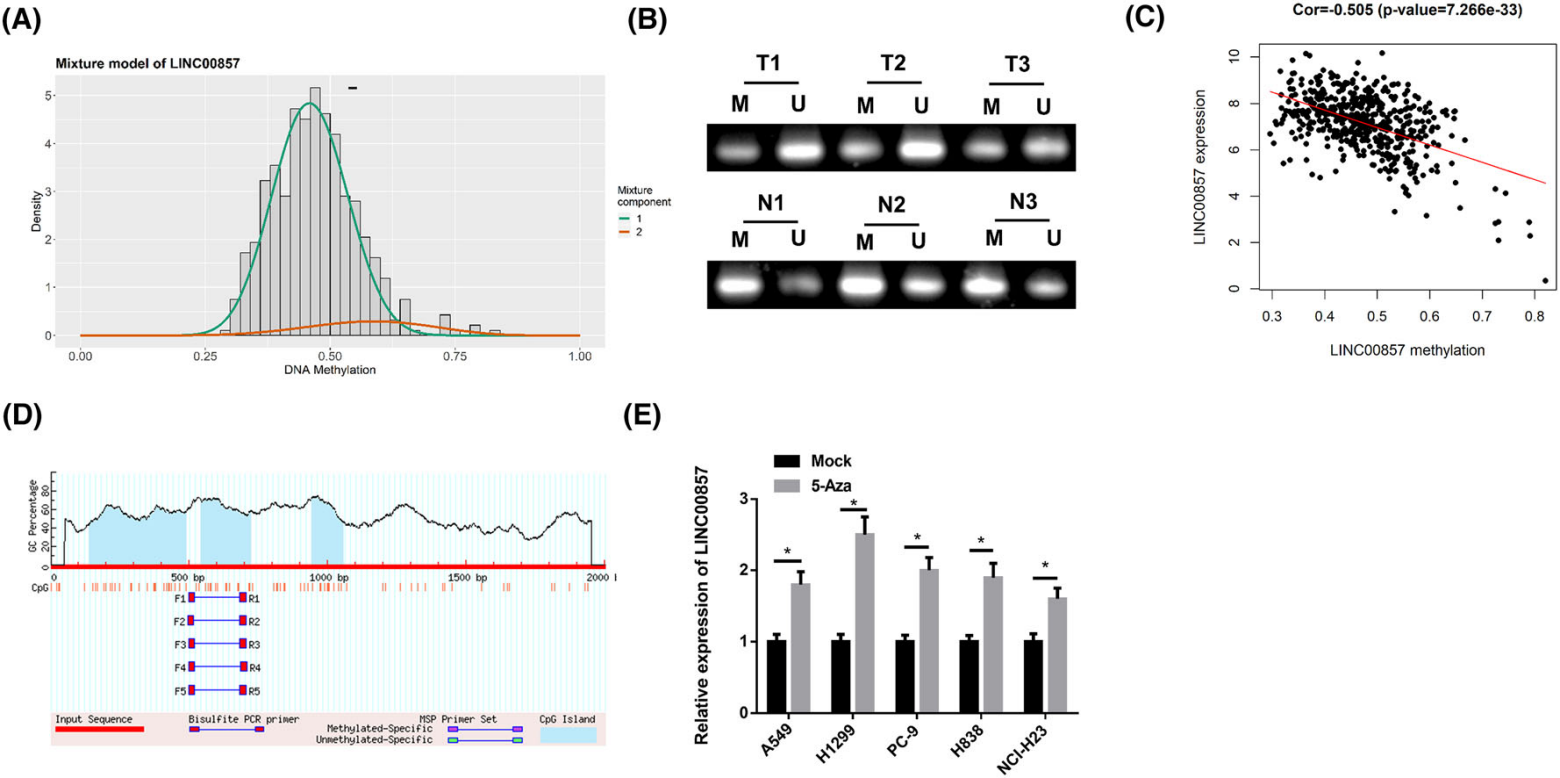
The upregulation of LINC00857 is due to hypomethylation in the promoter region. (A) The methyl mixed model of LINC00857. The distribution map represents the methylation status of methylated genes, the histogram shows the distribution of methylation in tumor samples, and the horizontal black bars indicate the distribution of methylation in normal samples; (B) MSP was used to detect the methylation level of LINC00857 in three pairs of paired LUAD tissue (T) and adjacent tissue (N) (M: methylated; U: unmethylated); (C) Pearson correlation analysis of LINC00857 methylation level and expression level; (D) the schematic structure of the CpG island in the LINC00857 promoter region predicted by the MethPrimer website; (E) qRT‐PCR was conducted to measure the expression changes of LINC00857 in different LUAD cells treated with 5‐Aza‐dC; **P < 0.01, ***P < 0.001.

Since DNA methylation is a common way of epigenetic modification that control gene expression, we attempted to predict methylation level of LINC00857 in LUAD tissue. Our findings uncovered that LINC00857 was hypomethylated in LUAD tissue (Figure 3A). LINC00857 methylation level in clinical LUAD and adjacent tissue was confirmed by Methylation‐specific PCR (MSP) analysis. The methylation level of LINC00857 in LUAD tumor tissue was prominently lower than in matched normal tissue (Figure 3B). Next, we detected a negative association between methylation level of LINC00857 and its expression through bioinformatics database (Figure 3C). In addition, we also detected the CpG island in LINC00857 promoter region through MethPrimer website (Figure 3D). After treating these LUAD cells with methyltransferase inhibitor 5‐Aza‐dC, LINC00857 level in LUAD cells was evidently upregulated (Figure 3E). Hence, these data suggest that the upregulation of LINC00857 in LUAD cells was due to the hypomethylation of the promoter region.

### 3.4 MiR‐486‐5p is the target of LINC00857 in LUAD

**FIGURE 4:**
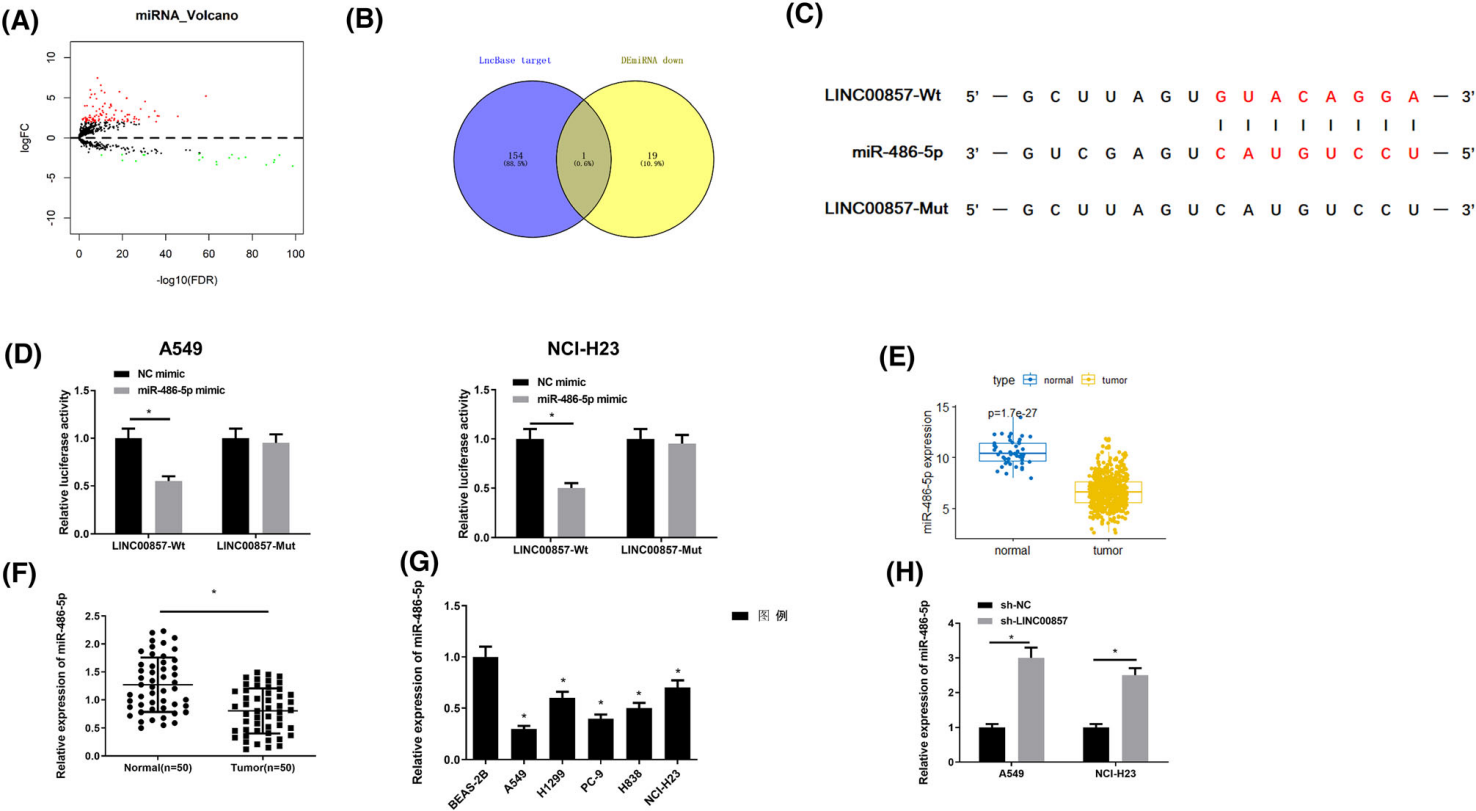
MiR‐486‐5p is the target of LINC00857 in LUAD. (A): Volcano map of the differential genes between the normal group and the tumor group in TCGA‐LUAD mature miRNA data set. The red dots represent the differentially upregulated genes and the green dots represent the differentially downregulated genes; (B) Venn graph of the target miRNAs and differential miRNAs of LINC00857 predicted by lncBase database; (C) the binding site of LINC00857 and miR‐486‐5p predicted by the lncBase database; (D) the binding site of LINC00857 and miR‐486‐5p detected by the dual‐luciferase reporter gene assay; (E): box plot of the expression of miR‐486‐5p in the LUAD tumor group and normal group in the TCGA‐LUAD database; (F) expression of miR‐486‐5p in clinical LUAD tissue and paired adjacent tissue; (G) the expression of miR‐486‐5p in human normal lung epithelial cell line BEAS‐2B and human LUAD cell lines; (H) the effect of sh‐NC or sh‐LINC00857 treatment on the expression of miR‐486‐5p; **P < 0.001, ***P < 0.001, ****P < 0.0001.

To unveil possible regulatory mechanism of LINC00857, we intersect 20 downregulated miRNAs, obtained from the differential analysis, and the target miRNA of LINC00857, predicted by the lncBase database, to obtain miR‐486‐5p (Figure 4A,B). The lncBase database also predicted binding sites of LINC00857 into miR‐486‐5p (Figure 4C). Furthermore, dual‐luciferase gene analysis indicated that, compared with cells transfected with pmirGLO‐LINC00857‐MUT and miR‐486‐5p mimic, luciferase activity was substantially reduced in cells with pmirGLO‐LINC00857‐WT and miR‐486‐5p mimic (Figure 4D). Differential analysis of miR‐486‐5p level in LUAD and normal tissue in TCGA‐LUAD unraveled that miR‐486‐5p level in tumor tissue was evidently reduced (Figure 4E). We also tested miR‐486‐5p level in LUAD tissue and cells, uncovering marked low levels (Figure 4F,G). The effect of knockdown of LINC00857 on miR‐486‐5p level was assayed in cells via qRT‐PCR. Knockdown of LINC00857 caused a substantial increase in miR‐486‐5p level (Figures 4H and 1B). Together, LINC00857 negatively modulated miR‐486‐5p level in the cytoplasm through targeted binding.

### 3.5 LINC00857 affects malignant progression of LUAD cells

**FIGURE 5:**
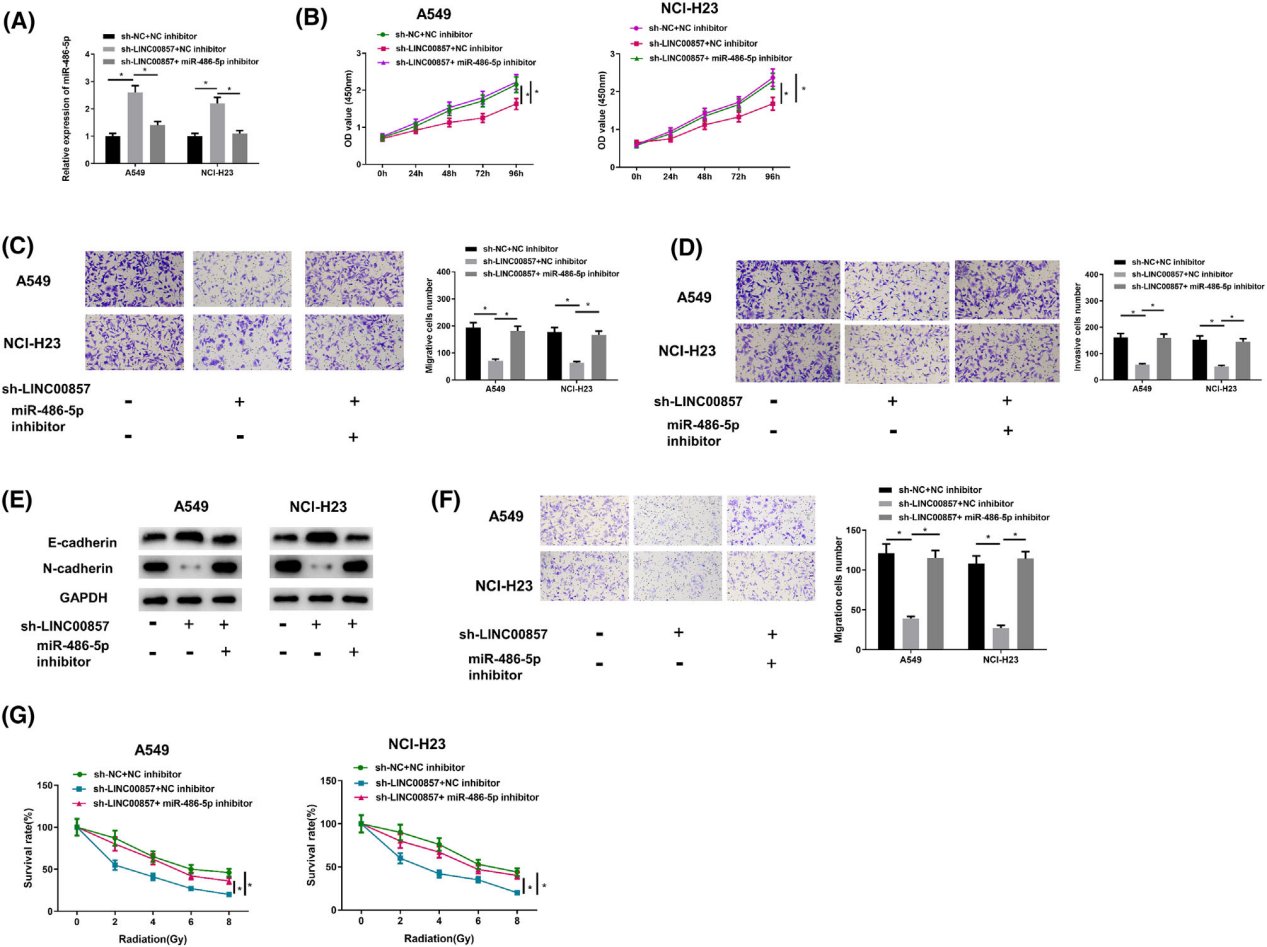
LINC00857 affects malignant progression of LUAD cells. (A) qRT‐PCR detected the expression level of miR‐486‐5p in LUAD cells after different transfection treatments; (B) CCK‐8 analyzed the viability of LUAD cells after different transfection treatments; (C, D) Transwell analyzed the migration and invasion of LUAD cells after different transfection treatments (100×); (E) Western blot detected the expression of EMT markers E‐cadherin and N‐cadherin in LUAD cells after different transfection treatments; (F) representative graphs and quantification of Transwell migration assay in HLECs cultured in medium supernatant of the abovementioned cells (100×); (G) the proliferative activity of LUAD cells in each transfection group detected after different intensities of X‐ray radiation treatment; *P < 0.05, **P < 0.01, ***P < 0.0001.

In an effort to investigate involvement of miR‐486‐5pin malignant behavior of LUAD cells mediated by LINC00857, three groups of transfected cells were set up: sh‐NC + NC inhibitor, sh‐LINC00857 + NC inhibitor, sh‐LINC00857 + miR‐486‐5p inhibitor system. Then, miR‐486‐5p level in A549 and NCI‐H23 cells was tested. Knockdown of LINC00857 enhanced miR‐486‐5p level, and miR‐486‐5p inhibitor + sh‐LINC00857 group exhibited a notable downregulation in miR‐486‐5p level (Figure 5A). Next, proliferation, migration, invasion, radiosensitivity, lymphocyte migration and lymphatic vessel formation of A549 and NCI‐H23 cells were examined. sh‐LINC00857 significantly suppressed proliferation (Figure 5B), migration and invasion (Figure 5C,D), EMT process (Figure 5E), and lymphocyte migration (Figure 5F). In addition, knockdown of LINC00857 also decreased stimulated radiosensitivity (Figure 5G). In contrast, miR‐486‐5p inhibitor significantly rescued impact of LINC00857 knockdown on all above‐mentioned biological functions. These findings indicated that LINC00857 modulated the malignant phenotype of LUAD cells by sponging miR‐486‐5p.

### 3.6 LINC00857 modulates NEK2 expression through sponge adsorption of miR‐486‐5p

**FIGURE 6:**
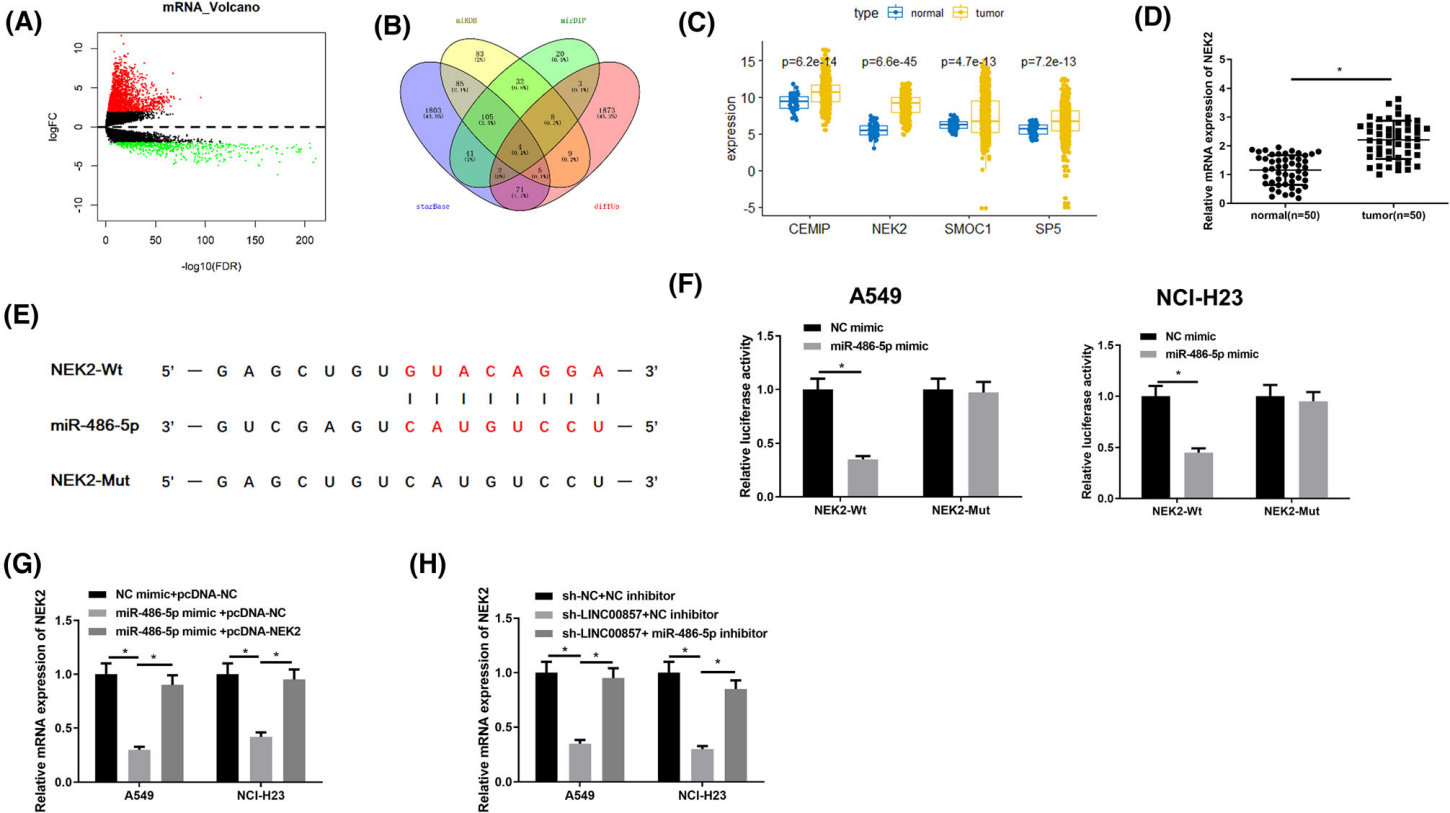
LINC00857 modulates NEK2 expression through sponge adsorption of miR‐486‐5p. (A) The volcano map of the differential genes between the normal group and the tumor group in the mRNA data set of TCGA‐LUAD, the red dots represent differentially upregulated mRNA, and the green dots represent differentially downregulated mRNA; (B) Venn diagram of possible target mRNAs and differential mRNAs of miR‐486‐5p predicted by starBase, miRDB, and mirDIP; (C) box plot of the expression levels of candidate target genes CEMIP, NEK2, SMOC1, and SP5 in the LUAD tumor group and the normal group; (D) mRNA expression level of NEK2 in 50 cases of clinical LUAD tissue and adjacent tissue; (E) binding sites of miR‐486‐5p and NEK2 predicted by starBase database; (F) dual‐luciferase reporter gene assay detected the binding relationship between miR‐486‐5p and NEK2; (G) qRT‐PCR detected the mRNA level of NEK2 in the transfected cells of the NC mimic + pcDNA‐NC, miR‐486‐5p mimic + pcDNA‐NC, and miR‐486‐5p mimic + pcDNA‐NEK23 groups; (H) qRT‐PCR detected the effect of LINC00857 and miR‐486‐5p on NEK2 transcription level; ***P < 0.001, ****P < 0.0001.

To study modulatory target genes downstream of miR‐486‐5p, upregulated mRNAs obtained from the differential analysis were overlapped with the possible target genes screened through databases. Four potential downstream targets of miR‐486‐5p were acquired, namely CEMIP, NEK2, SMOC1, and SP5 (Figure 6A,B). Among them, NEK2 presented the highest differential expression in LUAD tumor tissue (Figure 6C). Hence, we believed that NEK2 may be target downstream of miR‐486‐5p. On this basis, the NEK2 mRNA expression in LUAD tissue was detected, which was prominently boosted stacked up to normal tissue (Figure 6D). The starBase database expressed that miR‐486‐5p bound to 3′‐UTR of NEK2 (Figure 6E). Hence, we performed dual‐luciferase assay in vitro to evaluate whether miR‐486‐5p can bind to NEK2. MiR‐486‐5p mimic caused a prominent reduction in luciferase activity of WT NEK2 but had no obvious influence on that of MUT NEK2 (Figure 6F). We verified the regulatory impact of miR‐486‐5p on NEK2. A549 and NCI‐H23 cells were transfected with NC mimic + pcDNA‐NC, miR‐486‐5p mimic + pcDNA‐NC and miR‐486‐5p mimic + pcDNA‐NEK2, respectively, and the expression of NEK2 was determined at the transcription levels. As revealed by the results, forced expression of miR‐486‐5p constrained NEK2 mRNA, while NEK2 overexpression reversed significant reduction of NEK2 (Figure 6G). Finally, influence of LINC00857/miR‐486‐5p axis on NEK2 levels was also tested. The results confirmed that knockdown of LINC00857 downregulated NEK2 mRNA expression, and miR‐486‐5p inhibitor restored NEK2 expression (Figures 6H and S1C). As a result, we concluded that LINC00857 could positively modulate the expression of NEK2 by adsorbing miR‐329‐3p.

### 3.7 Upregulation of NEK2 rescues inhibitory impact of sh‐LINC00857 on cell malignant phenotype in LUAD

**FIGURE 7:**
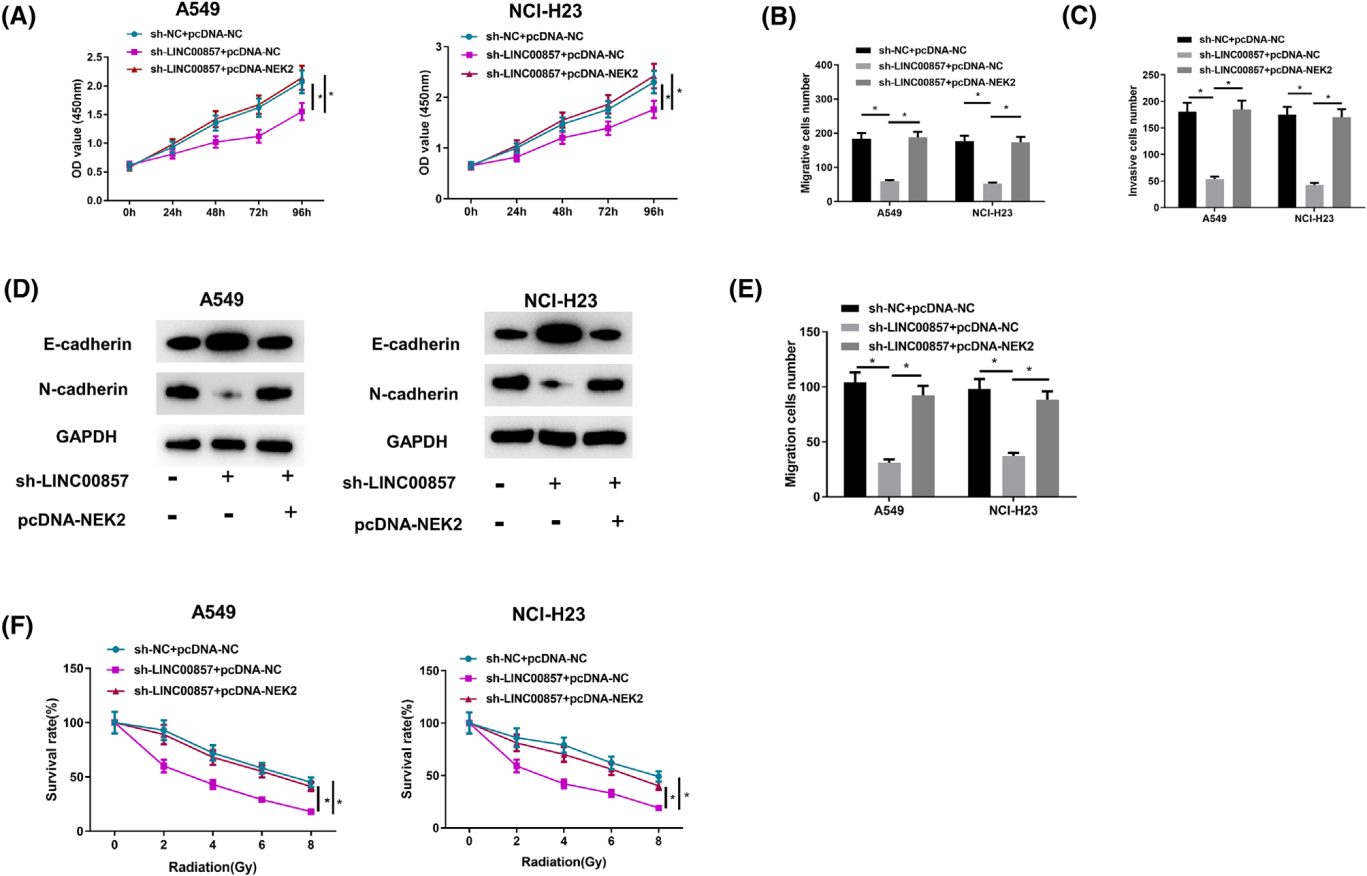
Upregulation of NEK2 reverses the inhibitory effect of sh‐LINC00857 on the malignant phenotype of LUAD cells. (A) CCK‐8 analyzed the viability of LUAD cells after different transfection treatments; (B, C) Transwell analyzed the migration and invasion of LUAD cells after different transfection treatments (100×); (D) Western blot detected the expression of EMT marker proteins E‐cadherin and N‐cadherin in LUAD cells after different transfection treatments; (E) representative graphs and quantification of Transwell migration assay in HLECs cultured in medium supernatant of the abovementioned cells; (F) the proliferative activity of A549 and NCI‐H23 cells in each transfection group detected after different intensities of X‐ray radiation treatment; *P < 0.05, **P < 0.01, ***P < 0.001.

Since NEK2 expression is modulated via LINC00857/miR‐486‐5p, it was believed that NEK2 may also participate in sh‐LINC00857‐mediated LUAD inhibition. To verify this, A549 and NCI‐H23 cells were co‐transfected with sh‐LINC00857 and pcDNA‐NC. Proliferative activity, migration and invasion were substantially declined in sh‐LINC00857 + pcDNA‐NC group, with radiosensitivity increased noticeably. In HLECs maintained in medium supernatant of these cells, lymphocyte migration was suppressed following knockdown of sh‐LINC00857 (Figure 7A–F). NEK2 participated in inhibitory influence of sh‐LINC00857 on the malignant phenotype of LUAD cells.

### 3.8 Knockdown of LINC00857 represses growth of LUAD tumor in vivo

**FIGURE 8:**
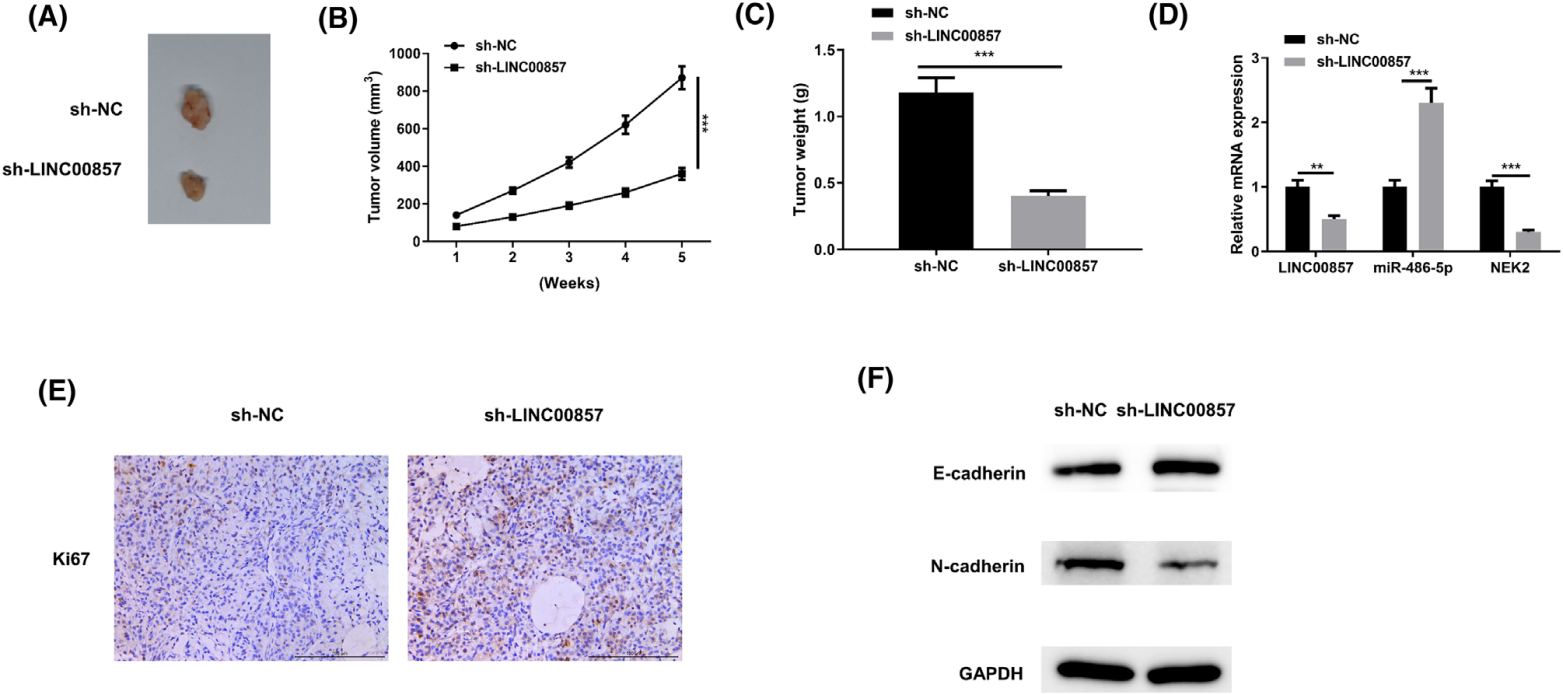
Knockdown of LINC00857 represses the growth of LUAD tumor in vivo*.* (A) Pictures of subcutaneous tumor xenografts collected from mice in the sh‐LINC00857 and sh‐NC groups; (B) tumor volume growth curve; (C) tumor weight 5 weeks after xenotransplantation; (D) qRT‐PCR determined the effect of sh‐LINC00857 on the expression of LINC00857, miR‐486‐5p and NEK2 in tumor tissues; (E) the expression of Ki67 in xenograft tumors was detected by immunohistochemistry. (F) Western blot detected the expression of EMT markers E‐cadherin and N‐cadherin in tumor tissues. **P < 0.01, ***P < 0.001.

To unveil influence of LINC00857 on tumor growth in vivo, A549‐sh‐LINC00857 cells were injected subcutaneously into limbs of nude mice. Tumor volumes were measured weekly and volume curves were plotted. Following euthanasia, tumor tissues were excised and representative images of subcutaneous tumor xenografts were taken 5 weeks after cell inoculation (Figure 8A). Records of tumor volume and weight revealed that they were markedly lowered in sh‐LINC00857 group than the sh‐NC group (Figure 8B,C). LINC00857, miR‐486‐5p and NEK2 levels in LUAD tissues of nude mice were detected. MiR‐486‐5p level in sh‐LINC00857 group was evidently accelerated, while LINC00857 and NEK2 mRNA expression was remarkably reduced in comparison to sh‐NC group, (Figure 8D). In addition, the low expression of LINC00857 could further enhance the sensitivity of LUAD cells to radiation (Figure S3). In summary, LINC00857 knockdown repressed LUAD tumor growth in vivo.

## 4 DISCUSSION

As molecular transcription research developed, gene expression has stood out in lung cancer research.^23^ DNA methylation is a critical epigenetic mechanism modulating gene expression, and its abnormal changes have become a common event in tumorigenesis.^24^, ^25^ Recent studies showed that DNA methylation is related to transcription disorder of lncRNA genes in cancer. Dong et al. disclosed that the downregulation of lncRNA MEG3 in esophageal cancer is related to abnormal hypermethylation of the MEG3 promoter.^26^ SSTR5 expression is restrained in laryngeal squamous cell carcinoma to play an anti‐tumor effect, and its expression is modulated by DNA methylation and histone modification.^27^ SOX21‐AS1 and SOX21 levels in oral squamous cell carcinoma were downregulated by DNA hypermethylation.^28^ HUMT level in triple‐negative breast cancer was upregulated due to promoter hypomethylation.^29^ We uncovered that LncRNA LINC00857 was substantially increased as mediated by hypomethylation of its promotor in LUAD. A CpG island in LINC00857 promoter region was predicted through the MethPrimer website.

LINC00857 exerts oncogenic effect in varying cancers through promoting cell proliferation, invasion and migration. For example, LINC00857 knockdown in esophageal adenocarcinoma reduced cell proliferative, invasive and migratory capabilities and induces apoptosis.^30^ In hepatocellular carcinoma cells, LINC00857 knockdown stimulates apoptosis and constrains cell cycle progression in G1 phase. In addition, it also regulates the EMT process to repress hepatocellular carcinoma cell phenotype.^31^ LINC00857 also plays a role in promoting pancreatic cancer.^32^ These studies have shown that LINC00857 may be function as an oncogene. A study provides evidence of substantial high expression of LINC00857 in LUAD^33^, which is in line with our results. Based on the database, bioinformatics methods are used to preliminarily investigate the expression of genes and analyze whether genes can be used for prognosis and diagnosis. This is a common way,^34^, ^35^ which is essential for subsequent experiments. The expression of genes in this study is determined based on bioinformatics methods and subjected to follow‐up experiment. Our results demonstrated that knockdown of LINC00857 markedly reduced LUAD proliferative activity, migratory and invasive capabilities, as well as lymph node metastasis and increased radiosensitivity. In addition, we also confirmed the effects of LINC00857 in promoting tumor growth in nude mice. Overall, these findings indicated that LINC00857 may represent an encouraging oncogenic lncRNA in the progression of LUAD.

LINC00857 was mainly located in cytoplasm, proposing that it may serve as a ceRNA in LUAD. Previous study suggested that expression changes of lncRNA‐encoding genes associated with methylation changes impact its downstream targets.^6^ MiR‐486‐5p was predicted as a downstream target of LINC00857 in LUAD through a series of bioinformatics analysis. Moreover, miR‐486‐5p mimic notably lowered luciferase activity of LINC00857‐WT in A549 and NCI‐H23 cells. Interaction of miR‐486‐5p and LINC00857 was then confirmed through qRT‐PCR, which displayed a negative correlation between their expressions in LUAD. Further functional studies also determined that miR‐486‐5p participated in LUAD suppression mediated by LINC00857 knockdown.

MiR‐486‐5p is a general genomic deletion region. These regions contain possible tumor repressors in varying tumors.^36^ MiR‐486‐5p is deemed as a master regulator involved in driving cancer initiation.^37^, ^38^, ^39^ Hence, bioinformatics analyses were performed to predict the targets of miR‐486‐5p. NEK2, a downstream target of miR‐486‐5p, presented highest differential expression in tumor tissue. Bioinformatics prediction exhibited that 3′‐UTR of NEK2 had a binding site into miR‐486‐5p. These findings made us believe that NEK2 was a possible target downstream of miR‐486‐5p. In an earlier study, miR‐486‐5p is remarkably underexpressed in LUAD cells, repressing their proliferative, migratory, and invasive properties.^40^ Our results were the same as the above‐mentioned investigations.

Previous report demonstrated that, NEK2 is necessary to maintain the transformed phenotype of cancer cells and regulate the transforming growth, survival and chemoresistance of tumors.^41^ Targeting NEK2 is a promising treatment for lung cancer.^42^ Wang et al.^43^ revealed that transcription of NEK2 in C2CD4D‐AS1 exerts a stimulative impact on LUAD cell malignant phenotype. In this study, we did functional tests by co‐transfecting sh‐LINC00857 and pcDNA‐NEK2 into LUAD cells. It was proved that forced expression of NEK2 reversed repressive impact of sh‐LINC00857 on LUAD cell proliferation, migration, invasion, lymph node metastasis, and the stimulative effect on radiosensitivity.

Taken together, LINC00857 was substantially upregulated, in LUAD tissue and cells, owing to hypomethylation in the promoter region. LINC00857 Knockdown constrains in vitro proliferation, migration, invasion, and lymph node metastasis of LUAD cells, and impairs in vivo tumor growth. LINC00857 could function as an oncogenic regulator through miR‐486‐5p/NEK2 axis. In conclusion, our findings indicate that LINC00857 plays an important role in the malignant progression of LUAD. LINC00857 has the potential as a therapeutic target, which may be used to guide clinicians in the diagnosis and prognosis of LUAD. At the same time, LINC00857/miR‐486‐5p/NEK2 axis may provide ideas for the clinical treatment of LUAD. Our study enriches the mechanism of LUAD. Of course, expanding the sample size in future research and deepening our research are important directions for our research.

## AUTHOR CONTRIBUTIONS

Haoyu Fu conceived and designed the study. Mingming Zhang and Xiaohui Liu performed the experiments. Yiming Yang and Ying Xing wrote the paper. Haoyu Fu reviewed and edited the manuscript. All authors read and approved the manuscript.

## CONFLICT OF INTEREST STATEMENT

The authors declare no conflicts of interest.

## ETHICS STATEMENT

The project was approved by the Ethics Committee of Tangshan People's Hospital (No. RMYY‐LLKS‐2023‐107) and Experimental Animal Ethics Committee of North China University of Technology (No. LX‐2012405). Informed consent was obtained from all patients.

## Supporting information


**Figure S1.** The expression of LINC00857 affects the expression of miR‐486‐5p and NEK2. A: qRT‐PCR was used to detect the expression of LINC0085 in LUAD cells of different treatment groups; B: qRT‐PCR was used to detect the expression of miR‐486‐5p5 in LUAD cells of different treatment groups; C: qRT‐PCR was used to detect the expression of NEK2 in LUAD cells of different treatment groups; *** *P* < 0.001, **** *P* < 0.0001.

**Figure S2.** Overexpression of LINC00857 promotes malignant progression of LUAD cells. A: A549 and NCI‐H23 cells were transfected with oe‐NC or oe‐LINC00857, and the transfection efficiency was detected by qRT‐PCR; B: CCK‐8 assay was used to detect the viability of A549 and NCI‐H23 cells transfected with oe‐NC or oe‐LINC00857; C‐D: Transwell assay was applied to detect the migration and invasion of A549 and NCI‐H23 cells transfected with oe‐NC or oe‐LINC00857 (100×); E: Western blot was employed to detect the effect of up‐regulation of LINC00857 on the expressions of EMT markers E‐cadherin and N‐cadherin; F: Different intensities of X‐ray radiation treatment was used for proliferation determination of A549 and NCI‐H23 cells transfected with oe‐NC or oe‐LINC00857; ** *P* < 0.01, *** *P* < 0.001.

**Figure S3.** Knockdown of LINC00857 enhances LUAD radiosensitivity in vivo*.* A:Flow chart of radiation therapy in xenograft mice；B: Pictures of subcutaneous tumor xenografts collected from mice in the sh‐LINC00857 + 4 Gy, sh‐NC + 4Gy and sh‐NC groups; C: Tumor volume growth curve; D: Tumor weight 25 days after xenotransplantation; D: The expression of Ki67 in xenograft tumors was detected by immunohistochemistry. ****P < 0.0001.

**Table S1.** Details of methylation data obtained based on TCGA database.

**Table S2.** Details of miRNA expression data obtained based on TCGA database.

**Table S3.** Details of mRNA expression data obtained based on TCGA database.

**Table S4.** Screened methylation driver genes.

## Data Availability

The data and materials in the current study are available from the corresponding author on reasonable request.
