# Supplementary figures and images for: Abnormal methylation mediated upregulation of LINC00857 boosts malignant progression of lung adenocarcinoma by modulating the miR‐486‐5p/NEK2 axis
Source: Clin Respir J. 2024 May 9;18(5):e13765. doi: 10.1111/crj.13765 (PMC11079885; doi:10.1111/crj.13765)

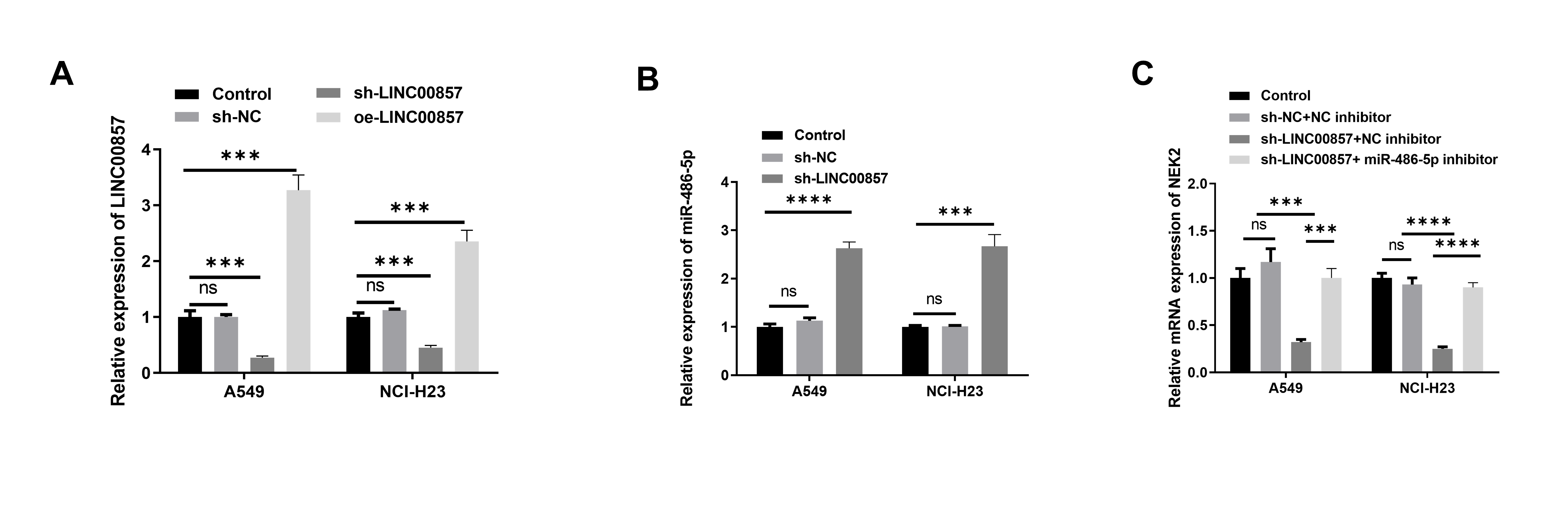

Supplement: Supplementary file 1 — Figure S1. The expression of LINC00857 affects the expression of miR‐486‐5p and NEK2. A: qRT‐PCR was used to detect the expression of LINC0085 in LUAD cells of different treatment groups; B: qRT‐PCR was used to detect the expression of miR‐486‐5p5 in LUAD cells of different treatment groups; C: qRT‐PCR was used to detect the expression of NEK2 in LUAD cells of different treatment groups; *** P < 0.001, **** P < 0.0001. [file CRJ-18-e13765-s004.tif]

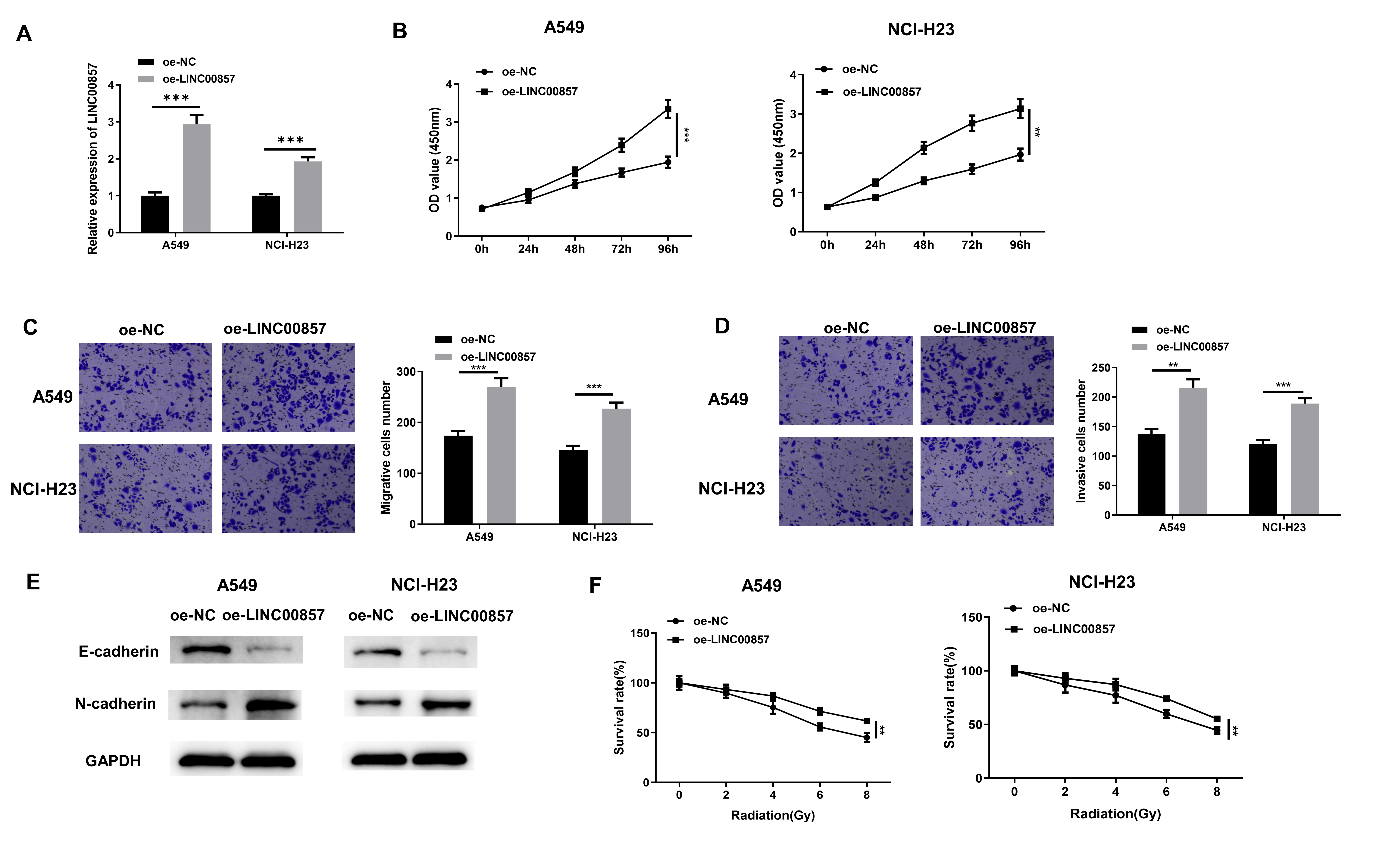

Supplement: Supplementary file 2 — Figure S2. Overexpression of LINC00857 promotes malignant progression of LUAD cells. A: A549 and NCI‐H23 cells were transfected with oe‐NC or oe‐LINC00857, and the transfection efficiency was detected by qRT‐PCR; B: CCK‐8 assay was used to detect the viability of A549 and NCI‐H23 cells transfected with oe‐NC or oe‐LINC00857; C‐D: Transwell assay was applied to detect the migration and invasion of A549 and NCI‐H23 cells transfected with oe‐NC or oe‐LINC00857 (100×); E: Western blot was employed to detect the effect of up‐regulation of LINC00857 on the expressions of EMT markers E‐cadherin and N‐cadherin; F: Different intensities of X‐ray radiation treatment was used for proliferation determination of A549 and NCI‐H23 cells transfected with oe‐NC or oe‐LINC00857; ** P < 0.01, *** P < 0.001. [file CRJ-18-e13765-s005.tif]

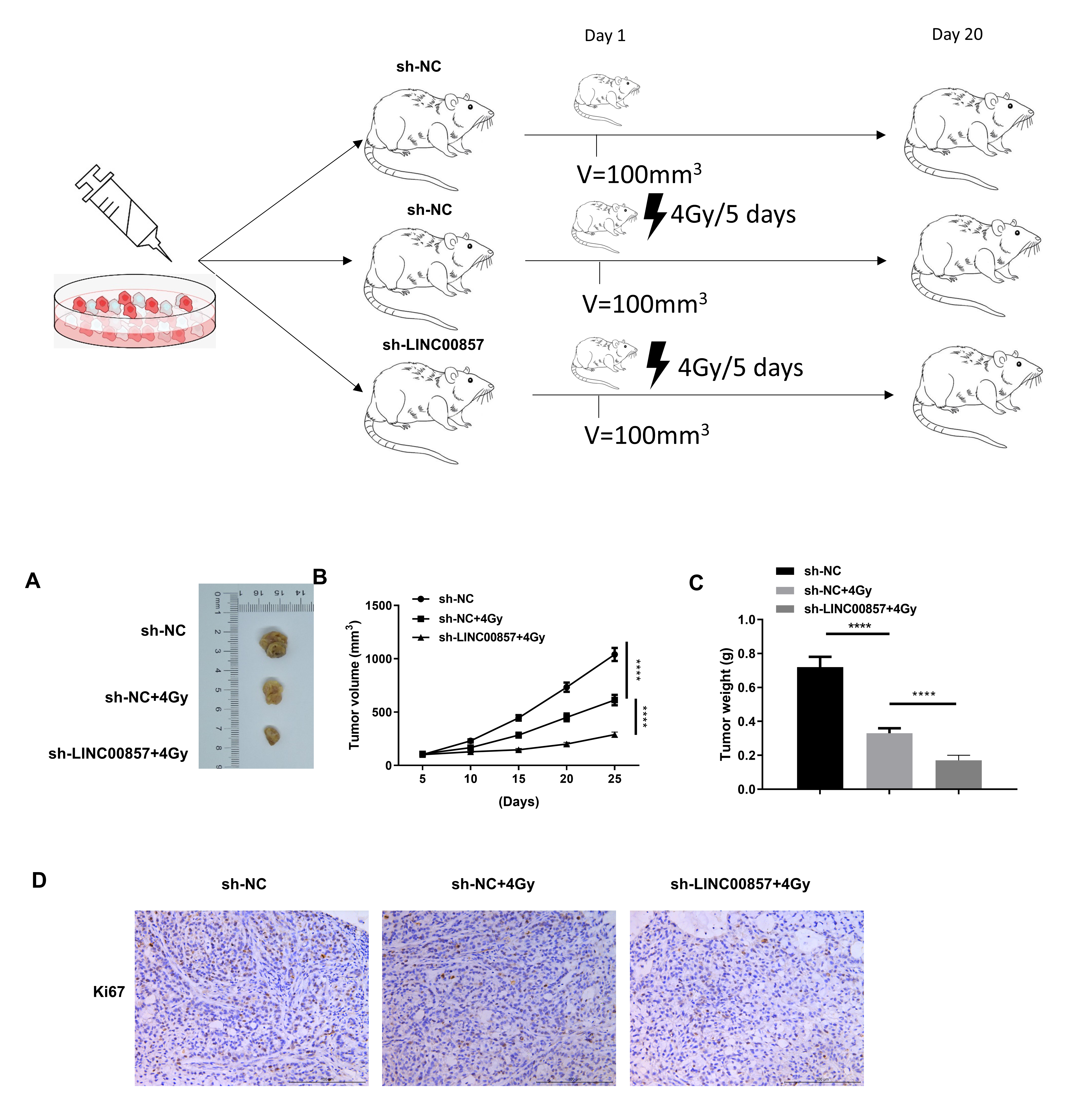

Supplement: Supplementary file 3 — Figure S3. Knockdown of LINC00857 enhances LUAD radiosensitivity in vivo. A:Flow chart of radiation therapy in xenograft mice；B: Pictures of subcutaneous tumor xenografts collected from mice in the sh‐LINC00857 + 4 Gy, sh‐NC + 4Gy and sh‐NC groups; C: Tumor volume growth curve; D: Tumor weight 25 days after xenotransplantation; D: The expression of Ki67 in xenograft tumors was detected by immunohistochemistry. ****P < 0.0001. [file CRJ-18-e13765-s007.tif]
